# Medicaid Expansion and Overall Mortality Among Women With Breast Cancer

**DOI:** 10.1001/jamanetworkopen.2025.54512

**Published:** 2026-01-27

**Authors:** Oluwasegun Akinyemi, Oladayo Oyebanji, Mojisola Fasokun, Faith Abodunrin, Chioma Ekwunazu, Fadeke Ogunyankin, Babawale Oluborode, Kakra Hughes, Miriam Michael, Robin Williams, Shari Lawson

**Affiliations:** 1 The Clive O. Callender Outcomes Research Center, Howard University College of Medicine, Washington, DC; 2 Department of Internal Medicine, University Hospitals Cleveland Medical Center, Cleveland, Ohio; 3 Department of Epidemiology, University of Alabama at Birmingham; 4 Department of Hematology and Oncology, University of Chicago, Chicago, Illinois; 5 Department of Research Data Science and Analytics, Cook Children’s Health Care System: Cook Children’s Medical Center, Fort Worth, Texas; 6 Department of Surgery, Henry Ford Hospital, Detroit, Michigan; 7 Department of Surgery, Howard University College of Medicine, Washington, DC; 8 Department of Obstetrics and Gynecology, Howard University College of Medicine, Washington, DC

## Abstract

**Importance:**

Medicaid expansion under the Patient Protection and Affordable Care Act (ACA) was designed to improve access to care and reduce health disparities. Its association with breast cancer mortality and related disparities remains unclear.

**Objectives:**

To evaluate the association between Medicaid expansion and overall mortality among women with breast cancer and to assess whether survival differs by race and ethnicity, disease stage, income, and treatment modality.

**Design, Setting, and Participants:**

This retrospective, hospital-based cohort study used a National Cancer Database cohort of 1 595 845 women aged 40 to 64 years with breast cancer who received a diagnosis from January 1, 2006, to December 31, 2021. States were classified as either Medicaid expansion or nonexpansion states. Statistical analyses were performed between January and July 2025.

**Exposure:**

Residence in a state that implemented Medicaid expansion by January 2014.

**Main Outcomes and Measures:**

The primary outcome was all-cause mortality. Policy effects were derived from difference-in-differences Cox proportional hazards regression models, reported as hazard ratios (HRs) and percentage change in hazard. Effect modification by race and ethnicity, disease stage, zip code–level income, and treatment was tested with 3-way interactions and joint Wald χ^2^ tests.

**Results:**

Of 1 595 845 women with breast cancer (mean [SD] age, 53.7 [6.8] years), 922 862 (57.8%) lived in early-expansion states, and 672 983 (42.2%) in nonexpansion states. Medicaid expansion was associated with lower overall mortality (HR, 0.95; 95% CI, 0.95-0.96; *P* < .001), a 4.8% relative hazard reduction vs nonexpansion. Hazard reductions were −3.4% (95% CI, −4.2% to −2.6%) for non-Hispanic White women, −4.3% (95% CI, −6.3% to −2.2%) for non-Hispanic Black women, and −19.0% (95% CI, −20.8% to −17.2%) for Hispanic women; associations for non-Hispanic women of other race or ethnicity were not significant. Lower mortality was most pronounced among patients with metastatic disease (−13.9%; 95% CI, −20.0% to −7.2%), those in the highest-income neighborhoods (−9.7%; 95% CI, −10.7% to −8.7%), and those receiving immunotherapy (−24.1%; 95% CI, −28.6% to −19.3%).

**Conclusions and Relevance:**

The findings of this cohort study suggest that Medicaid expansion under the ACA was associated with lower overall mortality among women aged 40 to 64 years with breast cancer. Benefits were uneven, underscoring persistent racial and ethnic and socioeconomic disparities and the need for targeted interventions.

## Key Points

**Question:**

Is Medicaid expansion under the Patient Protection and Affordable Care Act associated with lower overall mortality among women with breast cancer?

**Findings:**

In a cohort study of 1 595 845 women aged 40 to 64 years with breast cancer, Medicaid expansion was associated with lower overall mortality. Lower mortality was observed across disease stage, race and ethnicity, neighborhood income, and treatment strata, with the largest relative gains among Hispanic women and with smaller gains among non-Hispanic Black women and residents of low-income areas.

**Meaning:**

The findings of this study suggest that Medicaid expansion was associated with improved survival among women with breast cancer, although the benefits were not equitably distributed.

## Teaser

This cohort study of more than 1.5 million women in the National Cancer Database evaluates the association between Medicaid expansion and overall mortality among women with breast cancer and assesses whether survival differs by demographic characteristics.

## Introduction

Breast cancer remains the most commonly diagnosed cancer and the second leading cause of cancer-related death among women in the US,^1,2,3^ surpassed only by lung cancer.^4^ Recent estimates suggest that in 2025, approximately 316 950 new cases of invasive breast cancer and 59 080 cases of ductal carcinoma in situ will be diagnosed, with an estimated 42 170 deaths expected from the disease.^5,6,7^ Although advances in early detection and treatment have improved survival overall, these benefits have not been equitably distributed across all populations.^8,9,10^

The economic burden of breast cancer is substantial, with the disease accounting for the highest treatment costs among all cancers, at $29.8 billion in 2020 alone.^11,12^ These costs are borne not only by patients and their families but also by the broader health care system.^13^ Despite progress in care, disparities persist.^14^ Black women have higher breast cancer mortality despite lower incidence, often presenting with later-stage disease.^15,16,17,18^ Social determinants of health, including insurance status, income, geography, and systemic racism, exacerbate these inequities and contribute to gaps in diagnosis, treatment, and survivorship care.^19,20,21,22,23,24^

To address such disparities, the Patient Protection and Affordable Care Act (ACA) was enacted in 2010,^25^ with Medicaid expansion serving as a key provision to improve health care access for low-income populations.^26^ States that implemented Medicaid expansion broadened eligibility to adults earning up to 138% of the federal poverty level, enabling millions to access preventive services and timely cancer care.^27^ Previous studies have demonstrated that Medicaid expansion was associated with earlier cancer diagnoses and improved treatment rates and outcomes for certain malignant neoplasms, including cervical, colorectal, and breast cancers.^28,29,30^

Although numerous studies have examined the associations of Medicaid expansion with breast cancer outcomes, most were conducted during the initial years after ACA implementation and may not have captured long-term trends in mortality or the durability of improvements in access and care.^31^ Moreover, relatively few studies have comprehensively assessed how Medicaid expansion is associated with disparities in survival by race and ethnicity and neighborhood-level income, particularly using robust statistical frameworks such as 3-way interaction models.^32^

To address these gaps, the present study uses a large national dataset and more than a decade of follow-up to evaluate the long-term association of Medicaid expansion with overall mortality among women with breast cancer. A key component of our analysis is the use of 3-way interaction terms to evaluate whether Medicaid expansion was associated with reductions in racial and ethnic and zip code–level income disparities in breast cancer survival. To better understand the policy’s mechanisms, we examined both the total association of Medicaid expansion (unadjusted for stage and treatment) and the direct association (adjusted for these mediators) with breast cancer outcomes. By leveraging a difference-in-differences (DiD) approach and stratified modeling, we offer a comprehensive assessment of how this major policy intervention is associated with breast cancer outcomes across diverse population subgroups.

## Methods

### Study Design and Data Source

We conducted a retrospective cohort study using the National Cancer Database (NCDB),^33,34^ a hospital-based registry maintained by the American College of Surgeons Commission on Cancer, which captures incident cancers from Commission on Cancer–accredited facilities across the US and includes patient demographic characteristics, tumor characteristics, treatment, and vital status. Consistent with the NCDB’s scope, the cohort is not population based and therefore reflects outcomes among patients treated at participating facilities. Analyses adhered to the Strengthening the Reporting of Observational Studies in Epidemiology (STROBE) reporting guideline for observational studies. University Hospitals Cleveland Medical Center determined that, because the study used deidentified NCDB data, it was deemed exempt from institutional review board oversight and informed consent was not required. All study procedures adhered to the NCDB data use agreement and ethical guidelines for research using secondary health data.

### Patient Population

We identified women aged 40 to 64 years with histologically confirmed breast cancer diagnosed between January 1, 2006, and December 31, 2021. Women older than 64 years were excluded to minimize crossover with universal Medicare eligibility. Patients with missing key variables were excluded in the primary complete-case analysis. The timing of Medicaid expansion exposure was assigned using the NCDB variable that classifies patients according to the Medicaid expansion status of their state of residence (expansion vs nonexpansion), including an indicator for early expansions aligned with the 2014 rollout. eTable 2 in Supplement 1 lists the states in each category and the implementation year.

### Exposure Definition and Study Periods

The policy exposure was Medicaid expansion under the ACA. Expansion status was defined at the state level using the NCDB expansion-status variable. For the primary analysis, we combined states that implemented expansion between 2010 and 2013 (early expansion) with those that expanded in January 2014, defining them collectively as the expansion group. States that had not implemented Medicaid expansion at the time of the dataset release were categorized as nonexpansion, while states that adopted expansion after January 2014 were excluded from the analysis to maintain focus and minimize heterogeneity.

The prepolicy period was from 2006 to 2013, and the postpolicy period was from 2014 to 2021. In sensitivity analyses, we restricted the postpolicy period to 2015 to 2021 to allow for policy wash-in and account for minor timing variation among early adopters. We also repeated analyses splitting the expansion group into early expansion states (2010-2013) and January 2014 expansion states to examine whether timing differences were associated with results.

### Outcomes and Time Scale

The primary outcome was overall mortality. Survival time was measured in years since diagnosis, with time origin at the date of cancer diagnosis and censoring at death or last contact. We report the median follow-up using the reverse Kaplan-Meier method and describe censoring patterns by expansion status.

### Covariates

In this study, we harmonized NCDB racial and ethnic categories into 4 mutually exclusive groups: Hispanic (any race), non-Hispanic Black, non-Hispanic White (reference), and other (American Indian or Alaska Native, Asian or Pacific Islander, or race coded as other or unknown). Race and ethnicity were included as NCDB-defined variables to evaluate potential differences in access to care and mortality outcomes across demographic groups. A prespecified set of covariates included age (continuous), zip code–level median household income (quartiles; quartile 4 was the highest income [reference]), insurance (private [reference], Medicaid, uninsured, other), facility type (academic, community, other), clinical stage at diagnosis (I-IV; stage I was the reference), Charlson-Deyo comorbidity score (0, 1, ≥2), and initial treatment modalities (surgery, chemotherapy, or immunotherapy).

### Statistical Analysis

Statistical analyses were performed between January and July 2025. We summarized baseline characteristics by Medicaid expansion status and ACA period using descriptive statistics (additional details of the analytic approach, sensitivity analyses, and state classifications are provided in eTable 2 in Supplement 1, which provides the state groupings and Medicaid expansion coding). The primary analysis estimated the association of Medicaid expansion with overall mortality using Cox proportional hazards regression models with a DiD specification: indicators for residence in an expansion state (state), post-ACA period (post), and their interaction (state × post). Baseline hazards were stratified by expansion status and year of diagnosis to align with DiD assumptions in survival settings, and robust standard errors were clustered at the facility level. Effects are reported as hazard ratios (HRs) and percentage change in hazard [(HR − 1) × 100] with 95% CIs.

Effect modification was prespecified and evaluated with 3-way interactions (state × post × race and ethnicity; state × post × disease stage; state × post × neighborhood income; and treatment strata). Subgroup-specific DiD effects were obtained from model-based marginal contrasts using balanced weighting and assessed with joint Wald χ^2^ tests.

To complement hazard-scale estimates, we estimated absolute differences in 5-year failure risk using Royston-Parmar flexible parametric survival models on the log cumulative hazard scale (*df* = 4) including state, post, and state × post, the same covariates, stratified baseline hazards (by expansion status and year), and facility-clustered robust standard errors. Postestimation margins with balanced weighting provided the 5-year failure probabilities under each state × post scenario; the DiD contrast on the probability scale was computed as [p(expansion, post) − p(expansion, pre)] − [p(nonexpansion, post) − p(nonexpansion, pre)], with 95% CIs via the delta method.

Assumptions and diagnostics included Schoenfeld residual tests (global and covariate specific) and log-log plots; where nonproportionality was suggested, time-varying effects (eg, interactions with log time) were estimated as sensitivity checks. Parallel trends were probed by restricting to prepolicy years and interacting year with expansion status; Kaplan-Meier curves stratified by expansion status provided descriptive context.

Sensitivity analyses addressed missing data and policy coding. First, we implemented multiple imputation with chained equations and refit the primary DiD Cox proportional hazards regression models, reporting pooled within-group posteffects vs preeffects and the DiD ratio. Second, we recoded policy exposure as (1) combined expansion (early adopters, 2010-2013, plus January 2014 adopters) vs nonexpansion and (2) split expansion (early vs January 2014), each contrasted with nonexpansion. Third, to allow for policy wash-in, we restricted the postperiod to from 2015 to 2020 (excluding 2014) and repeated analyses. Across specifications, we report within-group post-HRs vs pre-HRs and DiD HRs relative to nonexpansion.

Statistical significance for χ^2^ tests and Wald χ^2^ tests was set at 2-sided *P* < .05. Analyses were performed using Stata, version 16 (StataCorp LLC).

## Results

### Cohort and Baseline Characteristics

Of 1 595 845 women with breast cancer (mean [SD] age, 53.7 [6.8] years) in the NCDB, 740 850 received a diagnosis during the pre-ACA period (2006-2013) and 854 995 received a diagnosis during the post-ACA period (2014-2020) (Table 1). A total of 922 862 women (57.8%) lived in early-expansion states and 672 983 (42.2%) in nonexpansion states. Early-expansion states accounted for 58.5% of pre-ACA diagnoses and 57.2% of post-ACA diagnoses. Baseline characteristics were broadly similar across expansion groups within periods. Compared with the pre-ACA period, the post-ACA period showed a modest shift toward earlier stage at diagnosis (eg, in situ stage 0 increased from 21.7% to 23.7% in the early-expansion pre-ACA period and from 18.8% to 20.4% in the nonexpansion post-ACA period) and lower rates of uninsured individuals (4.6% in pre-ACA nonexpansion period vs 2.3% in pre-ACA early-expansion period; 4.1% in post-ACA nonexpansion period vs 1.3% in post-ACA early-expansion period). Comorbidity remained favorable (Charlson-Deyo score 0: 86.8%-88.9% in pre-ACA period; 85.4%-87.0% in post-ACA period).

**Table 1.** Baseline Demographic and Clinical Characteristics of Women With Breast Cancer in Early Medicaid Expansion vs Nonexpansion States

Characteristic	No. (%) of women in pre-ACA period (n = 740 850)		*P* value^a^	No. (%) of women in post-ACA period (n = 854 995)		*P* value^a^
	Nonexpansion states (n = 307 130 [41.5%])	Early expansion states (n = 433 720 [58.5%])		Nonexpansion states (n = 365 853 [42.8%])	Early expansion states (n = 489 142 [57.2%])	
Age, mean (SD), y	53.5 (6.8)	53.3 (6.8)	.49	54.1 (6.8)	54.0 (6.8)	.75
Race and ethnicity^b^						
Hispanic	34 136 (11.1)	51 123 (11.8)	<.001	37 863 (10.3)	53 414 (10.9)	<.001
Non-Hispanic Black	48 198 (15.7)	37 592 (8.7)		63 840 (17.4)	48 007 (9.8)	
Non-Hispanic White	216 111 (70.4)	315 859 (72.8)		248 216 (67.9)	339 698 (69.4)	
Other	8685 (2.8)	29 146 (6.7)		15 934 (4.4)	48 023 (9.8)	
Stage						
0 (In situ)	66 811 (21.7)	102 917 (23.7)	<.001	68 808 (18.8)	99 825 (20.4)	<.001
I	111 872 (36.4)	160 741 (37.1)		171 522 (46.9)	232 636 (47.6)	
II	78 284 (25.5)	103 351 (23.8)		70 072 (19.1)	88 360 (18.1)	
III	28 387 (9.2)	37 072 (8.5)		26 308 (7.2)	31 279 (6.4)	
IV	11 294 (3.7)	14 423 (3.3)		15 542 (4.2)	18 294 (3.7)	
Missing	10 482 (3.4)	15 216 (3.5)		13 601 (3.7)	18 748 (3.8)	
Insurance						
Not insured	14 040 (4.6)	9985 (2.3)	<.001	15 109 (4.1)	6550 (1.3)	<.001
Private	240 740 (78.4)	354 406 (81.7)		284 840 (77.9)	381 566 (78.0)	
Medicaid	23 738 (7.7)	39 678 (9.1)		26 682 (7.3)	62 925 (12.9)	
Medicare	21 684 (7.1)	25 863 (5.9)		29 663 (8.1)	33 023 (6.7)	
Other	6928 (2.3)	3788 (0.9)		9559 (2.6)	5078 (1.0)	
CCI						
0	266 668 (86.8)	385 492 (88.9)	<.001	312 441 (85.4)	425 589 (87.0)	<.001
1	33 911 (11.0)	40 509 (9.3)		40 683 (11.1)	49 233 (10.1)	
2	5110 (1.7)	6048 (1.4)		8393 (2.3)	9549 (1.9)	
≥3	1441 (0.5)	1671 (0.4)		4336 (1.2)	4771 (1.0)	
Income, $						
<38 000	48 155 (15.7)	32 451 (7.5)	<.001	60 798 (16.6)	38 743 (7.9)	<.001
38 000-47 999	61 186 (19.9)	51 938 (12.0)		75 176 (20.5)	62 971 (12.9)	
48 000-62 999	75 916 (24.7)	95 009 (21.9)		79 022 (21.6)	98 702 (20.2)	
≥63 000	94 510 (30.8)	221 250 (51.0)		97 866 (26.7)	227 076 (46.4)	
Missing	27 354 (8.9)	33 072 (7.6)		52 991 (14.5)	61 650 (12.6)	
Educational level, %^c^						
≥21.0	51 612 (16.8)	49 685 (11.5)	<.001	62 470 (17.1)	68 523 (14.0)	<.001
13.0-20.9	70 589 (23.0)	73 735 (17.0)		86 269 (23.6)	92 481 (18.9)	
7.0-12.9	70 979 (23.1)	110 174 (25.4)		91 066 (24.9)	131 841 (26.9)	
<7.0	86 633 (28.2)	167 160 (38.5)		73 090 (20.0)	134 736 (27.5)	
Missing	27 317 (8.9)	32 966 (7.6)		52 958 (14.5)	61 561 (12.6)	

Abbreviations: ACA, Patient Protection and Affordable Care Act; CCI, Charlson-Deyo Comorbidity Index.

^a^ Statistical reporting: all tests are 2-sided with α = .05.

^b^ Unless otherwise specified, Hispanic includes persons of any race, and non-Hispanic categories exclude individuals coded as Hispanic. Other includes individuals recorded in the National Cancer Database as American Indian or Alaska Native and Asian or Pacific Islander, as well as those with race coded as other or unknown.

^c^ Educational level is measured in the percentage of individuals in each census tract with a high school diploma or equivalent.

### Kaplan-Meier Survival Analysis

Kaplan-Meier adjusted survival curves (Figure) and multivariable survival models (eTable 7 in Supplement 1) demonstrated a modest but statistically significant mortality improvement in expansion compared with nonexpansion states after the ACA. The absolute 5-year reduction in overall mortality associated with Medicaid expansion was −1.4 percentage points (95% CI, −1.6 to −1.3 percentage points; *P* < .001). This difference corresponds to approximately 1400 deaths averted for every 100 000 patients with cancer gaining coverage over 5 years. In adjusted Cox proportional hazards regression models, mortality decreased significantly in expansion states after ACA implementation (HR, 0.95; 95% CI, 0.95-0.96; *P* < .001), whereas no significant change was observed in nonexpansion states.

**Figure.**
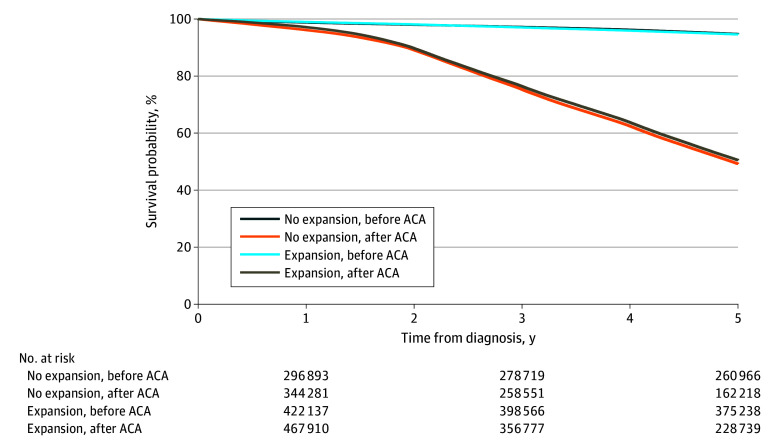
Kaplan-Meier Estimates of Overall Mortality Among Patients in Medicaid Expansion vs Nonexpansion States Before and After the Patient Protection and Affordable Care Act (ACA) The Kaplan-Meier curves display estimated overall survival comparing patients in Medicaid expansion vs nonexpansion states before and after implementation of the ACA. Over 5 years of follow-up, the adjusted difference-in-differences analysis demonstrated an absolute reduction in overall mortality of −1.4 percentage points (95% CI, −1.6 to −1.3 percentage points; Wald χ^2^_1_ = 273.89; *P* < .001) in expansion states compared with nonexpansion states. Estimates derive from Royston-Parmar flexible parametric survival models adjusted for demographic, clinical, and socioeconomic covariates.

### Primary DiD Effect by Race and Ethnicity

In Cox DiD models, Medicaid expansion was associated with a lower hazard of death overall (HR, 0.95; 95% CI, 0.95-0.96; −4.8% [−5.5% to –4.1%] change in hazard) (Table 2). By race and ethnicity, the DiD HR was 0.97 (95% CI, 0.96-0.97) for non-Hispanic White women (−3.4% [95% CI, −4.2% to −2.6%] change in hazard), 0.96 (95% CI, 0.94-0.98) for non-Hispanic Black women (−4.3% [95% CI, −6.3% to −2.2%] change in hazard), and 0.81 (95% CI, 0.79-0.83) for Hispanic women (−19.0% [95% CI, −20.8% to −17.2%] change in hazard). The HR estimate for non-Hispanic women of other race or ethnicity was 0.98 (95% CI, 0.95-1.02) and not statistically significant (χ^2^ = 0.97; *P* = .32). A joint Wald χ^2^ test indicated heterogeneity across racial and ethnic subgroups (χ^2^_4_ = 422.75; *P* < .001).

**Table 2.** DiD Estimates of Overall Mortality After Medicaid Expansion, by Race and Ethnicity

Race and ethnicity	DiD HR (95% CI)^a^^,^^b^	Change in hazard, %^c^	χ^2^ Value (*df*)	*P* value^b^
All	0.95 (0.95 to 0.96)	−4.8 (−5.5 to –4.1)	368.72 (1)	<.001
Hispanic	0.81 (0.79 to 0.83)	−19.0 (−20.8 to −17.2)	279.68 (1)	<.001
Non-Hispanic Black	0.96 (0.94 to 0.98)	−4.3 (−6.3 to −2.2)	15.68 (1)	<.001
Non-Hispanic White	0.97 (0.96 to 0.97)	−3.4 (−4.2 to −2.6)	64.37 (1)	<.001
Non-Hispanic Other	0.98 (0.95 to 1.02)	−1.8 (−5.2 to 1.8)	0.97 (1)	.32
Joint^d^	NA	NA	422.75 (4)	<.001

Abbreviations: ACA, Patient Protection and Affordable Care Act; DiD, difference-in-differences; HR, hazard ratio; NA, not applicable.

^a^ HRs estimated from Cox proportional hazards regression models with a state × ACA × race and ethnicity interaction and robust standard errors.

^b^ HRs of less than 1 indicate greater post-ACA mortality improvement in Medicaid expansion vs nonexpansion states.

^c^ Percentage change in hazard was calculated as (HR_DiD − 1) × 100.

^d^ Joint test reports heterogeneity across racial and ethnic subgroups.

### Heterogeneity by Stage at Diagnosis

Outcomes varied by stage at diagnosis (Table 3). The DiD HRs were 0.97 (95% CI, 0.96-0.99) for stage 0, 0.97 (95% CI, 0.96-0.98) for stage I, 0.99 (95% CI, 0.97-1.0) for stage II, 0.94 (95% CI, 0.91-0.96) for stage III, and 0.86 (95% CI, 0.80-0.93) for stage IV, corresponding to −2.8% (95% CI, −4.1% to −1.4%; *P* < .001), −3.0% (95% CI, −4.0% to −1.9%; *P* < .001), −1.4% (95% CI, −2.8% to −0.1%; *P* = .04), −6.4% (95% CI, −8.8% to −3.8%; *P* < .001), and −13.9% (95% CI, −20.0% to −7.2%; *P* < .001) changes in hazard, respectively. The joint test across stages was significant (χ^2^_5_ = 91.00; *P* < .001).

**Table 3.** DiD Estimates of Overall Mortality After Medicaid Expansion, by Disease Stage

Stage	DiD HR (95% CI)^a^^,^^b^	Change in hazard, %^c^	χ^2^ Value (*df*)	*P* value^b^
0 (In situ)	0.97 (0.96 to 0.99)	−2.8 (−4.1 to −1.4)	15.94 (1)	<.001
I	0.97 (0.96 to 0.98)	−3.0 (−4.0 to −1.9)	31.87 (1)	<.001
II	0.99 (0.97 to 1.00)	−1.4 (−2.8 to −0.1)	4.30 (1)	.04
III	0.94 (0.91 to 0.96)	−6.4 (−8.8 to −3.8)	23.43 (1)	<.001
IV	0.86 (0.80 to 0.93)	−13.9 (−20.0 to −7.2)	15.50 (1)	<.001
Joint^d^	NA	NA	91.00 (5)	<.001

Abbreviations: ACA, Patient Protection and Affordable Care Act; DiD, difference-in-differences; HR, hazard ratio; NA, not applicable.

^a^ HRs estimated from Cox proportional hazards regression models with a state × ACA × stage (STAGEB) interaction and robust standard errors.

^b^ HRs less than 1 indicate greater post-ACA mortality improvement in Medicaid expansion vs nonexpansion states.

^c^ Percentage change in hazard was calculated as (HR_DiD − 1) × 100.

^d^ Joint test reports heterogeneity across stage groups (STAGEB) using Wald χ^2^ statistics.

### Heterogeneity by Neighborhood Income

Neighborhood income modified the association between Medicaid expansion and cancer mortality (Table 4). The lowest-income quartile showed an increase in hazard (quartile 1: HR, 1.05 [95% CI, 1.03-1.07]; 4.8% [95% CI, 2.6%-7.1%] change in hazard), whereas quartiles 2 to 4 showed substantial reductions (quartile 2: HR, 0.91 [95% CI, 0.89-0.93]; quartile 3: HR, 0.89 [95% CI, 0.88-0.91]; and quartile 4: HR, 0.90 [95% CI, 0.89-0.91], corresponding to −9.1% [95% CI, −10.7% to −7.5%], −10.7% [95% CI, −12.0% to −9.4%], and −9.7% [95% CI, −10.7% to −8.7%] changes in hazard, respectively; all *P* < .001).

**Table 4.** DiD Estimates of Overall Mortality After Medicaid Expansion, by Neighborhood Income Quartile

Income quartile	DiD HR (95% CI)^a^^,^^b^	Change in hazard, %^c^	χ^2^ Value (*df*)	*P* value^b^
First	1.05 (1.03 to 1.07)	4.8 (2.6 to 7.1)	18.07 (1)	<.001
Second	0.91 (0.89 to 0.93)	−9.1 (−10.7 to −7.5)	116.50 (1)	<.001
Third	0.89 (0.88 to 0.91)	−10.7 (−12.0 to −9.4)	247.93 (1)	<.001
Fourth	0.90 (0.89 to 0.91)	−9.7 (−10.7 to −8.7)	318.82 (1)	<.001
Joint^d^	NA	NA	181.09 (4)	<.001

Abbreviations: ACA, Patient Protection and Affordable Care Act; DiD, difference-in-differences; HR, hazard ratio; NA, not applicable.

^a^ HRs estimated from Cox proportional hazards regression models with a state × ACA × neighborhood income quartile interaction and robust standard errors.

^b^ HRs less than 1 indicate greater post-ACA mortality improvement in Medicaid expansion vs nonexpansion states.

^c^ Percentage change in hazard was calculated as (HR_DiD − 1) × 100.

^d^ Joint test reports heterogeneity across neighborhood income quartiles.

### Heterogeneity by Treatment Strata

Results by treatment are presented in eTable 3 in Supplement 1. In brief, mortality reductions were larger among patients who received treatment. For surgery, the DiD HR was 0.95 (95% CI, 0.94-0.96; *P* < .001) with surgery vs 1.01 (95% CI, 0.96-1.06; *P* = .74) without surgery (joint χ^2^_2_ = 183.79; *P* < .001). For chemotherapy, the DiD HRs were 0.94 (95% CI, 0.93-0.96; *P* < .001) with chemotherapy vs 0.96 (95% CI, 0.95-0.97; *P* < .001) without chemotherapy (joint χ^2^_2_ = 168.92; *P* < .001). For immunotherapy, the largest association was observed among recipients (HR, 0.76; 95% CI, 0.71-0.81; *P* < .001) compared with nonrecipients (HR, 0.95; 95% CI, 0.94-0.96; *P* < .001) (joint χ^2^_2_ = 253.81; *P* < .001). Full estimates are reported in eTable 3 in Supplement 1.

### Supplementary Results and Sensitivity Analyses

The cohort flow diagram details sequential exclusions and the final analytic sample (eFigure 1 in Supplement 1). Prepolicy diagnostics show no evidence of differential trends between expansion and nonexpansion states from 2007 to 2013; yearly interaction terms are null, and the joint test is *F*_7,1197_ = 0.34 (*P* = .93) (eTable 1 and eFigure 2 in Supplement 1). State classifications and the Medicaid expansion coding used in all analyses are summarized in eTable 2 in Supplement 1. Stratified DiD Cox proportional hazards regression models show mortality reductions within treatment strata (surgery: HR, 0.95 [95% CI, 0.94-0.96]; change in hazard, −4.9 percentage points [95% CI, –5.6 to –4.2 percentage points]; and chemotherapy: HR, 0.94 [95% CI, 0.93-0.96]; change in hazard, −5.6 percentage points [95% CI, –6.6 to –4.5 percentage points]); the largest reduction was observed for immunotherapy (HR, 0.76 [95% CI, 0.71-0.81]; change in hazard, −24.1 percentage points [95% CI, –28.6 to –19.3 percentage points]), with significant joint tests across strata (eTable 3 in Supplement 1). Results are consistent when expansion is separated into early expansion vs January 2014 adopters (eTable 4 in Supplement 1) and remain robust when restricting the post-ACA period to 2015 and later (eTable 5 in Supplement 1). Multiple imputation sensitivity analyses corroborate complete-case findings: within-group post-ACA vs pre-ACA HRs were 19.3 (95% CI, 19.2-19.5) in nonexpansion and 18.9 (95% CI, 18.6-18.8) in expansion states, yielding a DiD ratio of 0.967 (95% CI, 0.961-0.974; *P* < .001) (eTable 6 in Supplement 1). On the absolute scale, DiD estimates of 5-year failure risk indicated an overall reduction of −1.4 percentage points (95% CI, −1.6 to −1.3 percentage points), with subgroup associations of −6.0 percentage points (95% CI, −6.6 to −5.5 percentage points) for Hispanic individuals, −0.1 percentage points (95% CI, −0.5 to 0.4 percentage points) for non-Hispanic Black individuals, −1.0 percentage points (95% CI, −1.2 to −0.8 percentage points) for non-Hispanic White individuals, and −2.2 percentage points (95% CI, –2.9 to –1.4 percentage points) for non-Hispanic individuals of other race or ethnicity and a significant joint test across subgroups (χ^2^_4_ = 582.37; *P* < .001) (eTable 7 in Supplement 1).

## Discussion

Using a hospital-based cohort of more than 1.5 million women with breast cancer, we found that Medicaid expansion under the ACA was associated with lower overall mortality across disease stage, race and ethnicity, neighborhood income, and treatment modality. The largest relative survival gains were seen among Hispanic women, those with advanced-stage disease, and residents of higher-income neighborhoods. Mortality also decreased among women treated with surgery, chemotherapy, or immunotherapy, with the greatest reduction in the immunotherapy subgroup. Despite broad improvements, reductions among non-Hispanic Black women and women of other race or ethnicity were small, and the 5-year relative decrease for non-Hispanic Black women was not statistically significant. These findings highlight population-wide benefits while underscoring persistent inequities; policies that pair coverage expansion with targeted efforts to improve timely diagnosis, treatment access, and adherence may be needed to close these gaps.

Building on prior national studies, which often combined multiple cancer types or concentrated on early-stage detection,^35,36^ our study offers a more focused evaluation of mortality trends among women with breast cancer. Our 7-year follow-up provides long-term insights beyond prior studies.^31^

Our findings complement and expand prior evidence by showing that Medicaid expansion was associated with improved survival across all disease stages and population strata, even after controlling for comorbidity and facility characteristics. The policy’s association with mortality varied across subgroups, with Hispanic women experiencing the greatest relative benefit and Black women continuing to face excess mortality risk despite overall survival gains.

To better understand the pathways through which Medicaid expansion is associated with breast cancer mortality, we conducted both unadjusted and adjusted DiD models. The primary models estimated the total association of Medicaid expansion with breast cancer mortality, capturing its clinical association, including earlier diagnosis and increased access to treatment. Sensitivity analyses that adjusted for cancer stage and treatment types yielded smaller but still significant survival benefits, reflecting the direct association of insurance coverage independent of care delivery. These complementary findings suggest that Medicaid expansion is associated with improved survival through multiple pathways, by facilitating timely detection, enabling treatment initiation, and reducing delays in care, underscoring the layered mechanisms through which policy interventions shape cancer outcomes.

Consistent with Lam et al,^37^ we observed lower mortality in Medicaid expansion states, associated largely with earlier diagnoses. However, unlike their study, in which mortality improvement was no longer evident after adjusting for disease stage, we continued to see significantly lower mortality even after accounting for disease stage and treatment. Medicaid expansion was associated with improved survival through early detection, treatment access,^37,38,39,40^ care continuity, and overall outcomes.^40,41,42^

These differences may reflect our exclusive focus on breast cancer, the inclusion of more recent data, or differences in state-level implementation of Medicaid expansion. In addition, our findings highlight nuances not previously reported, such as differential treatment access by income quartile and enhanced survival among those who received surgery and systemic therapies after expansion.

### Limitations

This study has some limitations. First, although a DiD design strengthens causal inference in observational research, residual and unmeasured confounding remains possible; factors such as patient-navigation services, local clinician capacity, and secular improvements in care may have been associated with outcomes despite adjustment. Second, socioeconomic status was measured at the area level (zip code–level median income) and may not capture individual deprivation, introducing potential ecological misclassification. Third, because data on cancer-specific mortality and recurrence were unavailable, we evaluated overall mortality; competing risks and cause-of-death misclassification may bias interpretation. Fourth, the observed stage-agnostic survival improvements may partly reflect contemporaneous gains in baseline health associated with Medicaid expansion, rather than outcomes confined solely to cancer care, which we were unable to fully disentangle. These findings support Medicaid expansion as a potentially lifesaving public health policy,^28,43^ particularly for women with breast cancer.^40,44,45,46^ Enhanced access to screening,^47^ early diagnosis,^39,48^ and timely treatment,^28^ especially in low-income and racially and ethnically diverse populations,^49^ were likely associated with observed survival gains. However, the persistence of racial and ethnic disparities, especially among Black women, despite coverage expansion^50,51^ suggests that insurance alone is insufficient and must be accompanied by targeted interventions to address structural racism,^52,53,54^ care fragmentation,^55,56,57^ and other social determinants of health.^54^

Further studies should investigate the mechanisms by which Medicaid expansion is associated with reduced mortality, including adherence to guideline-recommended therapies, continuity of care, and access to high-quality oncology services. Longitudinal research is also needed to determine whether the survival benefits associated with expansion persist over time and whether additional policy measures can eliminate residual disparities among Black women and other historically marginalized groups.

## Conclusions

In this large, hospital-based cohort study of women with breast cancer, Medicaid expansion under the ACA was associated with significant reductions in overall mortality across disease stages, racial and ethnic groups, income levels, and treatment types. Insurance expansion is associated with improved outcomes but must be paired with targeted efforts to address persistent disparities.

## Notes

Supplement 1. **eFigure 1.** Cohort Flow Diagram **eTable 1.** Pre-Policy Parallel-Trends Diagnostic (NCDB, 2007–2013) **eFigure 2.** Pre-Policy Mortality by Year and Expansion Status **eTable 2.** Medicaid Expansion State Groupings and Coding (NCDB, 2006–2021) **eTable 3.** Difference-in-Differences Estimates of Overall Mortality After Medicaid Expansion, by Treatment Strata **eTable 4.** Cox Regression by Early and January 2014 Expansion Groups **eTable 5.** Cox Regression by Early and January 2014 Expansion Groups (Post Period 2015+) **eTable 6.** Multiple Imputation Sensitivity Analysis: Post- vs Pre- ACA Effects and DID **eTable 7.** Difference-in-Differences (DiD) in 5-Year Failure Risk (Percentage Points): Overall and by Race/Ethnicity

Supplement 2.
Data Sharing Statement
