# Supplementary material for: Medicaid Expansion and Overall Mortality Among Women With Breast Cancer
Source: JAMA Netw Open. 2026 Jan 27;9(1):e2554512. doi: 10.1001/jamanetworkopen.2025.54512 (PMC12848628; doi:10.1001/jamanetworkopen.2025.54512)
Supplement: Supplement 1. — eFigure 1. Cohort Flow Diagram eTable 1. Pre-Policy Parallel-Trends Diagnostic (NCDB, 2007–2013) eFigure 2. Pre-Policy Mortality by Year and Expansion Status eTable 2. Medicaid Expansion State Groupings and Coding (NCDB, 2006–2021) eTable 3. Difference-in-Differences Estimates of Overall Mortality After Medicaid Expansion, by Treatment Strata eTable 4. Cox Regression by Early and January 2014 Expansion Groups eTable 5. Cox Regression by Early and January 2014 Expansion Groups (Post Period 2015+) eTable 6. Multiple Imputation Sensitivity Analysis: Post- vs Pre- ACA Effects and DID eTable 7. Difference-in-Differences (DiD) in 5-Year Failure Risk (Percentage Points): Overall and by Race/Ethnicity [file jamanetwopen-e2554512-s001.pdf]

Supplementary Online Content

Akinyemi O, Oyebanji O, Fasokun M, et al. Medicaid expansion and overall mortality among women with breast cancer. *JAMA Netw Open.* 2026;9(1):e2554512.
doi:10.1001/jamanetworkopen.2025.54512

eFigure 1. Cohort Flow Diagram

eTable 1. Pre-Policy Parallel-Trends Diagnostic (NCDB, 2007–2013)

eFigure 2. Pre-Policy Mortality by Year and Expansion Status

eTable 2. Medicaid Expansion State Groupings and Coding (NCDB, 2006–2021)

eTable 3. Difference-in-Differences Estimates of Overall Mortality After Medicaid Expansion, by Treatment Strata

eTable 4. Cox Regression by Early and January 2014 Expansion Groups

eTable 5. Cox Regression by Early and January 2014 Expansion Groups (Post Period 2015+)

eTable 6. Multiple Imputation Sensitivity Analysis: Post- vs Pre- ACA Effects and DID

eTable 7. Difference-in-Differences (DiD) in 5-Year Failure Risk (Percentage Points): Overall and by Race/Ethnicity

This supplementary material has been provided by the authors to give readers additional information about their work.

This supplement provides additional details and sensitivity analyses for the study “Medicaid Expansion and Overall Mortality in Women with Breast Cancer in the United States.” eFigure S1 shows the analytic cohort flow diagram. eTable S1 presents pre-policy parallel-trends diagnostics. eTable S2 details Medicaid expansion coding by state and study classification. eTable S3 reports difference-in-differences estimates of overall mortality by treatment strata. eTables S4 and S5 report Cox regression models by early and January 2014 expansion groups, including sensitivity analyses restricting the post period to 2015 and later. eTable S6 shows results from multiple imputation sensitivity analyses. eTable S7 presents absolute 5-year failure risk differences.

Abbreviations

ACA, Affordable Care Act; confidence interval (CI), confidence interval; DiD, difference-in-differences; hazard ratio (HR), hazard ratio; NCDB, National Cancer Database; pp, percentage points; standard error (SE), standard error; standard deviation (SD), standard deviation.

© 2026 Akinyemi O et al. *JAMA Network Open.*

eFigure 1. Cohort Flow Diagram

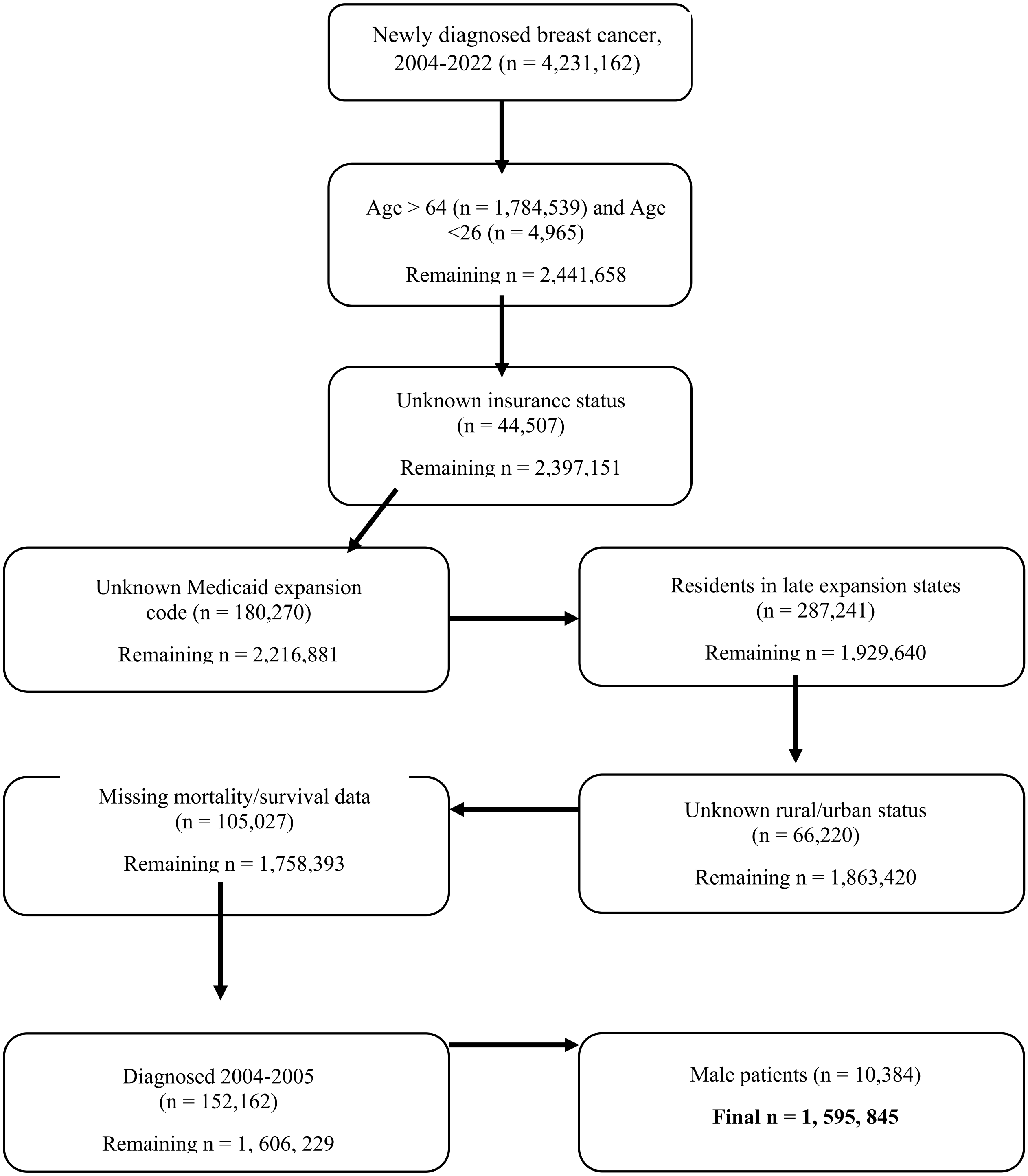

© 2026 Akinyemi O et al. *JAMA Network Open.*

eTable 1. Pre-Policy Parallel-Trends Diagnostic (NCDB, 2007–2013)

Year	Interaction coefficient, pp	95% CI, pp	P value
2007	-0.28	-1.15 to 0.58	.52
2008	0.22	-1.33 to 1.77	.78
2009	0.18	-1.37 to 1.74	.82
2010	0.25	-1.35 to 1.86	.76
2011	-0.01	-1.52 to 1.50	.99
2012	-0.23	-1.82 to 1.37	.78
2013	-0.17	-1.66 to 1.32	.82

Linear probability model of mortality including year fixed effects, an expansion indicator, and year×expansion interaction terms; standard errors clustered at the facility level. The analytic sample is restricted to pre-policy years (2007–2013). Reported coefficients are differences in mortality (percentage points) for expansion vs non-expansion by year (interaction terms). The joint test of all year×expansion interactions was $F(7, 1197) = 0.34$; $P = .93$, indicating no evidence of differential pre-policy trends.

Abbreviations: NCDB, National Cancer Database; pp, percentage points.

Model specification: mortality = year fixed effects + expansion indicator + year×expansion interactions; robust SEs clustered by facility (PUF_FACILITY_ID). The outcome is a binary mortality indicator; analysis limited to pre-policy years (2007–2013).

© 2026 Akinyemi O et al. *JAMA Network Open.*

eFigure 2. Pre-Policy Mortality by Year and Expansion Status

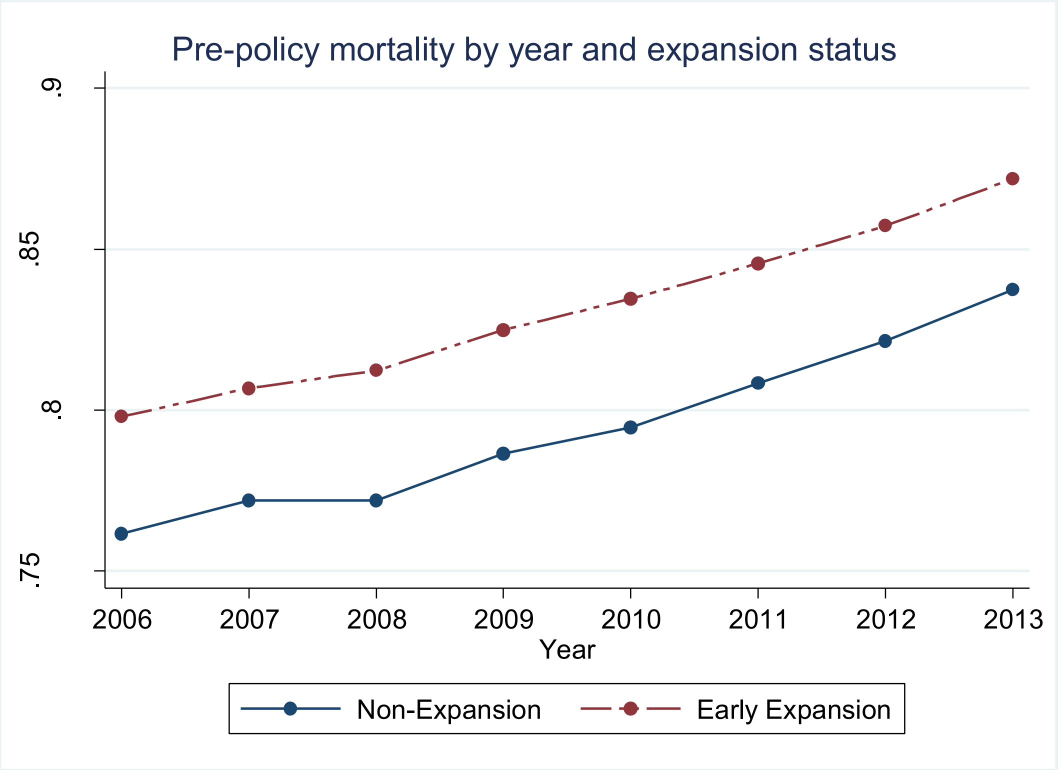

Both eTable 1 & eFigure 2 confirms that Parallel trend assumption holds for this study.

© 2026 Akinyemi O et al. *JAMA Network Open.*

eTable 2. Medicaid Expansion State Groupings and Coding (NCDB, 2006–2021)

Code	Definition	State Grouping
0	Non-Expansion States	TN, NC, ID, GA, FL, MO, AL, MS, KS, TX, WI, UT, SC, SD, VA, OK, NE, WY, ME
1	January 2014 Expansion States	KY, NV, CO, OR, NM, WV, AR, RI, AZ, MD, MA, ND, OH, IA, IL, VT, HI, NY, DE
2	Early Expansion States (2010–2013)	WA, CA, NJ, MN, DC, CT
3	Late Expansion States (after January 2014)	NH, IN, MI, PA, AK, MT, LA
9	Suppressed for Ages 0–39	Not available
(blank)	State Missing or Out of U.S.	Not available

Study Classification Used in Primary Analyses: Expansion states were Codes 1 and 2 (combined); non-expansion states were Code 0. Records with Codes 3 or 9 and missing/out-of-U.S. state were excluded from primary analyses. State groupings follow the National Cancer Database Medicaid expansion coding for 2006–2021. Abbreviations: NCDB, National Cancer Database; U.S., United States.

© 2026 Akinyemi O et al. *JAMA Network Open.*

eTable 3. Difference-in-Differences Estimates of Overall Mortality After Medicaid Expansion, by Treatment Strata

Stratum	Category	DiD HR (95 CI)	change in hazard	χ² (df)	P value
Surgery					
	None	1.01 (0.96–1.06)	+0.8 (-3.8 to +5.6)	0.11 (1)	.7433
	Surgery	0.95 (0.94–0.96)	-4.9 (-5.6 to -4.2)	183.68 (1)	<.001
	Joint	—	—	183.79 (2)	<.001
Chemotherapy					
	None	0.96 (0.95–0.97)	-4.0 (-4.9 to -3.1)	68.34 (1)	<.001
	Chemotherapy	0.94 (0.93–0.96)	-5.6 (-6.6 to -4.5)	100.77 (1)	<.001
	Joint	—	—	168.92 (2)	<.001
Immunotherapy					
	None	0.95 (0.94–0.96)	-4.9 (-5.6 to -4.2)	174.75 (1)	<.001
	Immunotherapy	0.76 (0.71–0.81)	-24.1 (-28.6 to -19.3)	79.05 (1)	<.001
	Joint	—	—	253.81 (2)	<.001

Hazard ratios (HRs) estimated from Cox proportional hazards models with an EXPAND × ACA × treatment stratum interaction and robust standard errors.

© 2026 Akinyemi O et al. *JAMA Network Open.*

eTable 4. Cox Regression by Early and January 2014 Expansion Groups

Group	DID HR (95% CI)	Change in hazard	χ^2 (df)	p-value
Non-Expansion	Reference			
Combined Expansion	0.95 (0.95–0.96)	-4.8 (-5.5 to –4.1)	40.34 (1)	<0.001
Early Expansion	0.98 (0.97–0.99)	-2.2 (-3.0 to -1.4)	25.52 (1)	<0.001
Jan 2014 Expansion	0.93 (0.93–0.94)	-6.7 (-7.4 to -5.9)	273.38 (1)	<0.001

Cox proportional hazards models with a difference-in-differences specification; robust standard errors. Adjusted for age, race/ethnicity, insurance, facility type, Charlson–Deyo comorbidity score, ZIP code–level income quartile, education quartile, and urbanicity.

© 2026 Akinyemi O et al. *JAMA Network Open.*

eTable 5. Cox Regression by Early and January 2014 Expansion Groups (Post Period 2015+)

Group	DID HR (95% CI)	change in hazard	χ^2 (df)	p-value
Non-Expansion	Reference			
Combined Expansion	0.96 (0.95–0.96)	-4.1 (-4.7 to –3.4)	131.80 (1)	<0.001
Early Expansion	0.98 (0.97–0.99)	-1.9 (-2.8 to –1.1)	18.49 (1)	<0.001
Jan 2014 Expansion	0.94 (0.93–0.95)	-6.0 (-6.8 to –5.2)	221.75 (1)	<0.001

Post period defined as 2015 and later (2014 excluded as wash-in). Cox proportional hazards models with a difference-in-differences specification; robust standard errors. Adjusted for age, race/ethnicity, insurance, facility type, Charlson–Deyo comorbidity score, ZIP code–level income quartile, education quartile, and urbanicity.

© 2026 Akinyemi O et al. *JAMA Network Open.*

eTable 6. Multiple Imputation Sensitivity Analysis: Post- vs Pre- ACA Effects and DID

Group / Contrast	Post vs Pre Ratio, exp(b) (95% CI)	P value
Non-Expansion (Post vs Pre)	19.32 (19.17–19.46)	<.001
Expansion (Post vs Pre)	18.68 (18.55–18.81)	<.001
DID Ratio (Expansion vs Non-Expansion)	0.967 (0.961–0.974)	<.001

Results are pooled across multiply imputed datasets using Rubin's rules. Multiple imputation via chained equations included demographic, clinical, and facility covariates, Medicaid expansion status (EXPAND), ACA period (POST), and the outcome. Estimates are hazard ratios [exp(b)] from Cox survival models including POST, EXPAND, and their interaction (EXPAND×POST), adjusted for prespecified covariates. Rows labeled "Post vs Pre" indicate within-group comparisons; the DiD ratio represents the interaction effect (EXPAND×POST).

Abbreviations: ACA, Affordable Care Act; DiD, difference-in-differences; HR, hazard ratio; CI, confidence interval.

© 2026 Akinyemi O et al. *JAMA Network Open.*

eTable 7. Difference-in-Differences (DiD) in 5-Year Failure Risk (Percentage Points): Overall and by Race/Ethnicity

Group	DiD, pp (95% CI, pp)	Wald χ^2 (df)	P Value
All individuals	−1.4 (−1.6 to −1.3)	273.89 (1)	<.001
Hispanic	−6.0 (−6.6 to −5.5)	450.10 (1)	<.001
Non-Hispanic Black	−0.1 (−0.5 to 0.4)	0.09 (1)	0.77
Non-Hispanic White	−1.0 (−1.2 to −0.8)	108.00 (1)	<.001
Other	-2.2 (-2.9 to -1.4)	31.56 (1)	<.001
Joint across subgroups	—	582.37 (4)	<.001

Abbreviations: ACA, Affordable Care Act; CI, confidence interval; DiD, difference-in-differences; pp, percentage points.

a. The "All individuals" row reports the overall absolute difference in predicted 5-year failure probability for post- vs pre-ACA in expansion vs nonexpansion states (Wald χ^2_1=273.89; P<.001).

b. Subgroup rows show race/ethnicity-specific DiD estimates with 95 CIs and corresponding Wald χ^2 tests (df=1).

c. The "Joint across subgroups" row reports a Wald test of equality across the four subgroup DiD estimates; no single estimate or CI is reported for this test.

d. Estimates derive from Royston–Parmar flexible parametric survival models on the hazard scale (df=4), adjusted for insurance, facility type, comorbidity, neighborhood income (quartiles), educational attainment (quartiles of without high school), and urbanicity, with robust standard errors. Results are presented as absolute risk differences (pp) in predicted 5-year failure probability.

© 2026 Akinyemi O et al. *JAMA Network Open.*
