# Supplementary material for: Medicaid Expansion and Overall Mortality Among Women With Breast Cancer
Source: JAMA Netw Open. 2026 Jan 27;9(1):e2554512. doi: 10.1001/jamanetworkopen.2025.54512 (PMC12848628; doi:10.1001/jamanetworkopen.2025.54512)
Supplement: Supplement 2. — Data Sharing Statement [file jamanetwopen-e2554512-s002.pdf]

Data Sharing Statement

Akinyemi. Medicaid Expansion and Overall Mortality Among Women With Breast Cancer. *JAMA Netw Open*. Published January 27, 2026. doi:10.1001/jamanetworkopen.2025.54512

Data
Data available: No

Additional Information
Explanation for why data not available: Data Availability: This study used data from the National Cancer Database (NCDB), a hospital-based registry jointly sponsored by the American College of Surgeons and the American Cancer Society. The NCDB is not publicly available due to data use agreements but may be accessed by eligible investigators through application to the American College of Surgeons. Data Access Requirements: Researchers seeking access to the NCDB must apply through the NCDB Participant User File (PUF) request system and meet eligibility criteria set by the Commission on Cancer. Supporting Documents: No additional documents (e.g., data dictionary, protocol) are publicly available. The dataset includes breast cancer cases diagnosed between January 1, 2006, and December 31, 2021. Restrictions: Per NCDB policy, data cannot be redistributed or shared publicly. Use is restricted to approved researchers and institutions. Supporting Documents: No additional documents (e.g., data dictionary, protocol) are publicly available. Data Sharing Timeline: Data access is ongoing through the NCDB PUF program. This study used data from the 2022 NCDB release (diagnosis years 2004-2022). Restrictions: Per NCDB policy, data cannot be redistributed or shared publicly. Use is restricted to approved researchers and institutions.
